# Nature of the Pits on the Lattice-Matched InAlAs Layer Surface Grown on the (001) InP Substrate

**DOI:** 10.3390/nano14221842

**Published:** 2024-11-18

**Authors:** Dmitrii V. Gulyaev, Demid S. Abramkin, Dmitriy V. Dmitriev, Alexander I. Toropov, Eugeniy A. Kolosovsky, Sergey A. Ponomarev, Nina N. Kurus, Ilya A. Milekhin, Konstantin S. Zhuravlev

**Affiliations:** 1 Laboratory of Molecular-Beam Epitaxy of A3B5 Compounds, Institute of Semiconductor Physics, SB RAS, Novosibirsk 630090, Russiaddmitriev@isp.nsc.ru (D.V.D.);; 2 Department of Physics, Novosibirsk State University, Novosibirsk 630090, Russia; 3 Engineering Department, Corporation “Novosibirsk Radio Components Factory “Oksid”, Novosibirsk 630102, Russia; 4 Laboratory of Optical Materials and Structures, Institute of Semiconductor Physics, SB RAS, Novosibirsk 630090, Russia; 5 Laboratory of Nanodiagnostics and Nanolithography, Institute of Semiconductor Physics, SB RAS, Novosibirsk 630090, Russia; 6 Near-Field Optical Spectroscopy and Nanosensor Laboratory, Institute of Semiconductor Physics, SB RAS, Novosibirsk 630090, Russia

**Keywords:** InAlAs, InP, molecular beam epitaxy, surface pits, solid alloy, desorption, surface morphology, threading dislocations

## Abstract

The structural properties of lattice-matched InAlAs/InP layers grown by molecular beam epitaxy have been studied using atomic force microscopy, scanning electron microscopy and micro-photoluminescence spectroscopy. The formation of the surface pits with lateral sizes in the micron range and a depth of about 2 ÷ 10 nm has been detected. The InP substrate annealing temperature and value of InAlAs alloy composition deviation from the lattice-matched In_x_Al_1−x_As/InP case (*x* = 0.52) control the density of pits ranging from 5 × 10^5^ cm^−2^ ÷ 10^8^ cm^−2^. The pit sizes are controlled by the InAlAs layer thickness and growth temperature. The correlation between the surface pits and threading dislocations has been detected. Moreover, the InAlAs surface is characterized by composition inhomogeneity with a magnitude of 0.7% with the cluster lateral sizes and density close to these parameters for surface pits. The experimental data allow us to suggest a model where the formation of surface pits and composition clusters is caused by the influence of a local strain field in the threading dislocation core vicinity on In adatoms incorporating kinetic.

## 1. Introduction

InAlAs layers lattice-matched with the InP are one of the main materials for microwave electronics and optoelectronics for the telecom wavelength range near 1.55 μm [1,2,3]. Such relevance for InAlAs is primarily due to the increased band gap for electrons as compared to phosphorus-containing structures, which increases the temperature stability and power of electronic devices with InAlAs barrier layers [4]. As a result, InAlAs layers are currently used in heterostructure designs for microwave field-effect transistors [5,6], lasers [7,8,9], electro-optical modulators [4,10], photodiodes [11] and photovoltaic cells [12,13].

Quite a large number of works, including the study of the defects in InAlAs layers, deal with the epitaxial growth of InAlAs layers on InP substrates [14,15,16,17,18,19,20,21,22,23]. However, most of the works focus on the study of structural defects in the bulk InAlAs [8,9,10,11,12,13,14,15,16,17,18,19,20,21]. The surface defects and mechanisms of their formation are much less studied. One of the surface defect types that can have a strong effect on the performance of many optoelectronic devices [7,22,24,25] is surface pits with diagonals along [110] directions [7,19,26]. In particular, it is shown that pits can affect the value of Schottky barriers formed on the InAlAs surface [24]. InGaAs/InAlAs/InP cascade lasers grown under conditions with minimal surface defects demonstrate lower threshold currents and improved efficiency [7]. Another important type of InAlAs/InP-based structures is heterostructures with InAs/InAlAs self-assembled quantum dots (SAQDs) widely used for laser fabrication in the telecom 1.55 μm wavelength range [1,2,3,27,28,29]. Surface morphology has a key role in SAQDs formation for a wide range of heterosystems [30,31,32,33,34,35,36,37,38,39,40,41], which makes morphology control essential.

The clarification of the surface pit formation mechanism is a necessary condition for InAlAs morphology control. This mechanism has not been finally established. The formation of pits on the surface of the InAlAs layer is usually attributed to the lower mobility of Al adatoms as compared to the mobility of Ga adatoms due to the absence of such surface defects in InGaAs layers. However, such an explanation does not take into account the different growth temperatures of InGaAs and InAlAs layers. The lowering of the growth temperature of InAlAs to the growth temperature of InGaAs leads to the disappearance of defects [7]. It allows us to suggest that the growth parameters, such as substrate temperature, have a crucial role in the formation of surface pits.

In this work, a detailed study of the surface morphology and elemental and structural composition of the InAlAs layer has been performed in order to determine the nature of the formation of pits on the surface of an InAlAs layer grown on an InP substrate.

## 2. Experiment

The investigated InAlAs layers were grown by molecular beam epitaxy (MBE) on semi-insulating epi-ready InP (001) substrates in a Riber Compact 21T setup. The preliminary step of oxide removal from the substrate was described in detail in [42]. It is necessary to note that the annealing procedure in the framework of substrate preparation results in the formation of a thin InAsP layer. The important parameter at this stage is substrate annealing temperature (*T*_A_). The subsequent growth of InAlAs layers on the atomically clean growth surface of the substrate was performed under excessive As_4_ pressure to suppress its desorption in the growth temperature range applied. The ratio of Group V to Group III fluxes was about 100 at the equivalent As_4_ pressure in the flux of *F_As*4*_* = 1.5–2.5 × 10^−5^ Torr. The layer growth rate determined by Group III flow was constant at all the growth temperatures (*T*_S_) and was equal to ~0.6 μm/h. The InAlAs growth was stopped once the layer thickness reached *D*. In situ temperature control for the substrate annealing and layer growth was carried out by readings of the infrared pyrometer “Ircon Modline Plus” calibrated by the temperature of reconstruction transitions on the InP surface [43]. The temperature measurement error was 1%. In situ, the control of the 2-dimensional growth of the InAlAs layer was carried out by reflection high-energy electron diffraction (RHEED) by the superstructure (2 × 4) typical of the As-enriched InAlAs surface.

**Figure 1:**
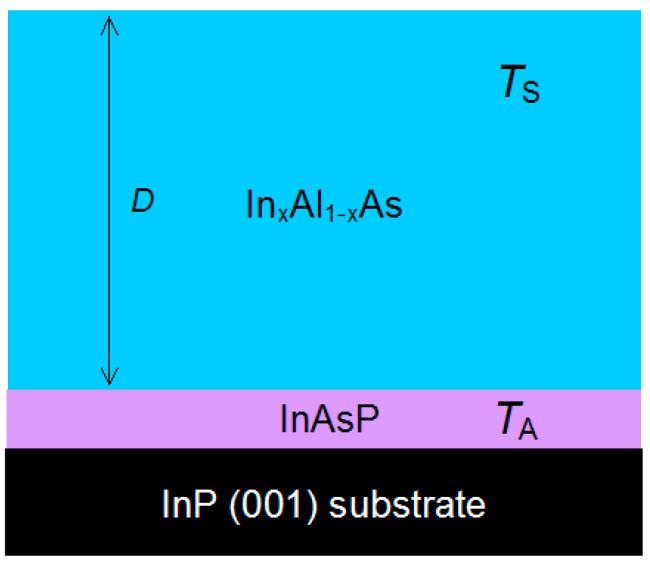
Scheme of structures with In_x_Al_1−x_As layer with thickness *D* grown at *T*_S_. The preparation of the InP substrate was performed at *T*_A_. The InAsP layer, formed during the substrate preparation, is marked as «InAsP».

In order to clarify the surface pit formation mechanism, the InAlAs/InP heterostructure series with variation in *T*_A_, *T*_S_ and *D* was grown. The parameter values were varied in the following ranges: *T*_A_ = 485 ÷ 550 °C, *T*_S_ = 485 ÷ 535 °C and *D* = 300 ÷ 1000 nm. Additionally, In_x_Al_1−x_As alloy composition deviated from the InP lattice-matched value. The structure series with alloy composition x ranging from 0.52 (lattice-matched) to 0.51 was grown. Other parameters for the series were *T*_A_ = 535 °C, *T*_S_ = 525 °C and *D* = 1000 nm. The scheme of structures is presented in Figure 1.

The surface morphology of InAlAs layers was determined using a Bruker Multimode 8 atomic force microscope (AFM). The structural analysis of the layers was performed by scanning electron microscopy (SEM) by HITACHI SU8220. The composition of the grown layers on the micro-scale was determined from the analysis of micro-photoluminescence (micro-PL) spectra. The micro-PL spectra were measured on a Horiba XPlorA setup. A 532 nm/0.1 mW wavelength/power laser was used for PL excitation. The focused spot size of the laser on the sample did not exceed 2 μm. The micro-PL map was measured with a step of 0.2 μm. To minimize the effects of Al oxidation, the samples were stored in an inert atmosphere.

## 3. Results

### 3.1. Atomic Force Microscopy

**Figure 2:**
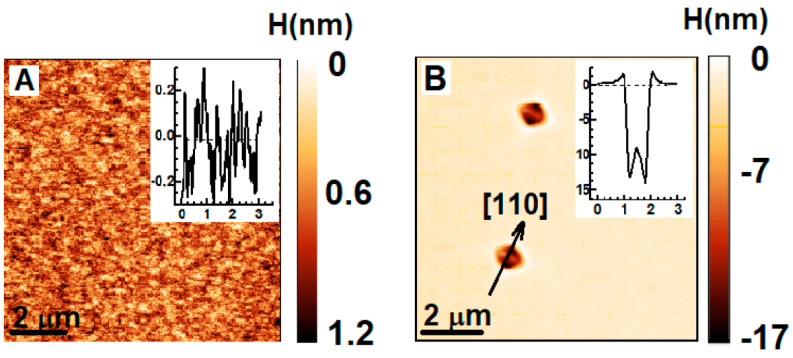
AFM images of 1 μm thick InAlAs layers lattice-matched with the substrate. *T*_A_ = 485 °C and *T*_S_ = 485 °C for (**A**) and *T*_A_ = 535 °C, *T*_S_ = 525 °C for (**B**). Total layer thickness is 1000 nm for both structures. Relief profiles are presented in the insets.

AFM images of the surface of InAlAs/InP lattice-matched layers grown at *T*_A_ = 485 °C and *T*_S_ = 485 °C (Figure 2A) and *T*_A_ = 535 °C, *T*_S_ = 525 °C (Figure 2B) are shown in Figure 2. The layer thickness *D* for both structures is about 1000 nm. One can see that at low temperatures, *T*_A_ and *T*_S_ (Figure 2A), there are no surface pits within the scanning area. The analysis of the large-scale images (about 20 × 20 μm^2^) also demonstrates the absence of surface pits. It allows us to conclude that the pit density at these growth conditions is lower than 3 × 10^5^ cm^−2^. In contrast, increasing the temperatures up to *T*_A_ = 535 °C and *T*_S_ = 525 °C results in the formation of surface pits with a density of about 2 × 10^6^ cm^−2^. The surface areas without pits are characterized by a root mean square roughness of about 0.2 nm for all the structures. It should be noted that the sizes and structure of the pits are characterized by great homogeneity in the framework of a single sample, although they are different in the case of various samples. The observed pits with depths up to several tens of nanometers are complete or truncated rhombs with their diagonals oriented along the [110] and [110] directions. The typical pit profiles are shown in the inset to the corresponding part of Figure 2B. As one can see, the pit profile has some peculiarities: (1) a ridge/barrier up to 1.5 nm high is formed along the perimeter of the complete rhombus-shaped pit, and (2) a small hillock up to a few nanometers high is formed in the center of the pits with a lateral size higher than ~1 μm.

**Figure 3:**
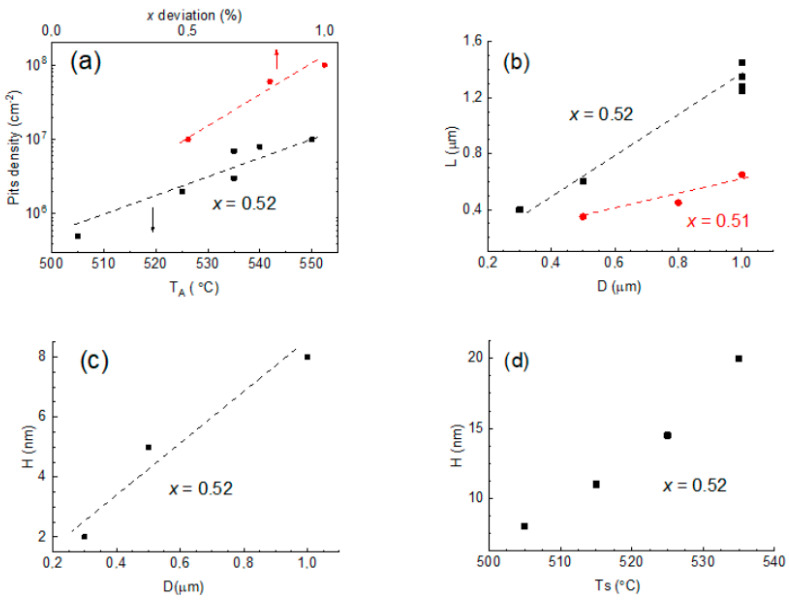
(**a**) The dependence of the pit density on the substrate annealing temperature for the lattice-matched layer (*x* = 0.52, black dots) and on the layer composition deviation from the lattice-matched value (red dots). (**b**) The dependence of the lateral size of the pits on the InAlAs layer thickness for *x* = 0.52 (black dots) and *x* = 0.51 (red dots). (**c**) The dependence of the depth of the pits on the thickness of the InAlAs layer with *x* = 0.52 grown at the temperature of 505 °C. (**d**) The dependence of the depth of the pits on the growth temperature for the InAlAs layer with *x* = 0.52 grown at the total layer thickness of 1 μm.

We analyzed AFM images for the structures grown at different growth conditions, which allowed us to obtain information about the dependencies of some crucial pit parameters (sizes and density) on the growth conditions. The dependencies of the pit density (*N*_P_) on *T*_A_ and the alloy deviation value are presented in Figure 3a. As one can clearly see from the figure, *N*_P_ strongly increases with *T*_A_, changing in the range of 505 ÷ 550 °C from 8 × 10^5^ cm^−2^ to 10^7^ cm^−2^. Furthermore, the alloy composition deviation from the lattice-matched value of 0.52 also results in an increase in *N*_P_ up to 10^8^ cm^−2^, as shown in Figure 3a. Importantly, *T*_S_ and *D* variations have no effect on the density of pits.

An increase in the InAlAs layer thickness *D* from 300 to 1000 nm in the case of the lattice-matched alloy composition leads to an increase in the lateral size of the pits (*L*) with the linear law as shown in Figure 3b (the black dots and line). A slight deviation of the alloy composition from the lattice-matched value of 0.5% has an effect on the *L* (D) dependence: the sizes decrease in general (about two times) and stay similar to a linear character of the dependence. Moreover, a rise in *D* results in an increase in the pit depth (*H*) with the near-linear law, as shown in Figure 3c. An increase in *T*_S_ also has an effect on *H*, and it monotonically rises with *T*_S_ from 505 °C to 535 °C, as one can see in Figure 3d.

### 3.2. Scanning Electron Microscopy

**Figure 4:**
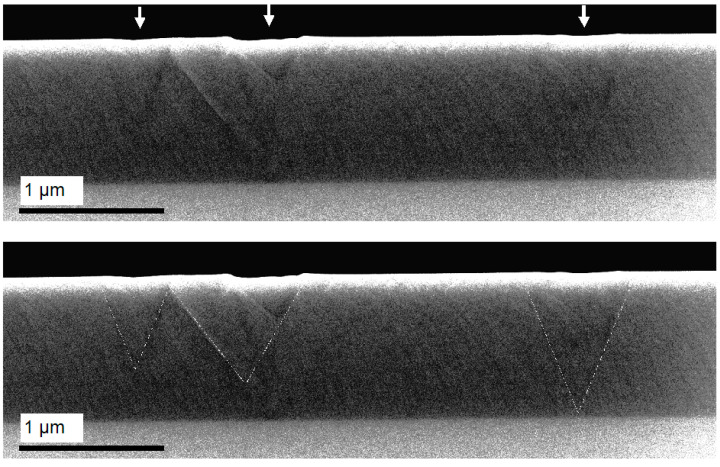
The SEM cross-sectional image of the heterostructure grown at *T*_A_ = 535 °C, *T*_S_ = 525 °C and *D* = 1000 nm, with the alloy composition deviation of 0.5%. The vertical arrows at the top panel point to the surface pits. The bottom panel shows the same area of the SEM image but with the dislocations indicated by thin white lines for better clarity.

To determine the structural perfection of the InAlAs layer under the pits [110], the cross-sections of the samples were examined by SEM. The SEM image of the heterostructure grown at *T*_A_ = 535 °C, *T*_S_ = 525 °C and *D* = 1000 nm, with the alloy composition deviation of 0.5 %, is presented in Figure 4. The figure clearly shows the threading dislocation in the InAlAs bulk and the surface pits correlating with the dislocation. There are two types of dislocations: (i) nucleated in the InAlAs/InP interface and (ii) nucleated in the bulk of the InAlAs layer. We associate the first type of dislocation with strain relaxation in the InAsP/InP thin layer [42] formed during the substrate preparation and the second one with strain relaxation into the InAlAs layer caused by the alloy composition deviation [18,19,20,21,44,45,46,47].

The main feature of the SEM image presented is that dislocation outcrops limit the pits along the perimeter and form a ridge on the surface. Importantly, the structure of pits obtained based on the SEM data is in good agreement with more precise AFM data on the pits. This pit–dislocation correlation was observed early in Ref. [18], where transmission electron microscopy images of InAlAs/InP layers were discussed.

### 3.3. Micro-Photoluminescence

**Figure 5:**
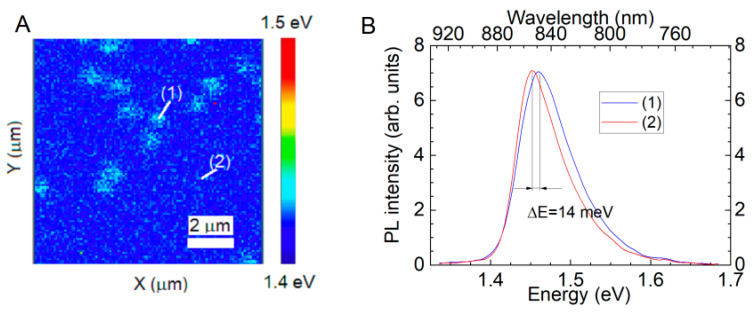
(**A**) A typical map of the position of the PL peak of the InAlAs layer with pits. (**B**) Micro-PL spectra from the InAlAs layer measured (1) inside and (outside) the cluster. The annealing temperature of the substrate/growth is 535/505 °C.

Room temperature μ-PL spectra for different InAlAs/InP heterostructures were measured, and maps of the distribution of the energy position of the interband transition were plotted. The spectra and map for a lattice-matched structure grown at *T*_A_ = 535 °C and *T*_S_ = 535 °C with *D* = 1000 nm are presented in Figure 5. PL spectra consist of a single band centered near 1.45 eV with a bandwidth of about 80 meV. The PL band is associated with the interband electron-hole optical transition in the InAlAs layer [7,48]. In our view, the broadening of the PL band is caused by low-scale InAlAs alloy inhomogeneity. As shown in Figure 5A, the peak position of the PL band is not constant over the InAlAs surface at the area of 10 × 10 μm. There are areas with the PL band peak shifted in a high-energy range by 14 meV in comparison with other areas. The main feature of these areas is the close values of the lateral size (about 1 μm) and density (10^6^ ÷ 10^7^ cm^−2^ for different structures) to the parameters of the surface pits observed in the AFM and SEM data. We associate the shift of the PL band peak energy with a change in the average InAlAs alloy composition in some areas compared with others. It is necessary to note that PL band peak inhomogeneity is observed for all the structures with surface pits, and the magnitude of the PL band peak energy shift increases with *T*_S_.

In order to estimate both the low-scale composition fluctuation magnitude and average composition change for the observed areas, we performed the calculation of the InAlAs band gap, taking into account the influence of the composition and elastic deformation caused by the composition deviation. The calculations were performed in the framework of the model-solid theory [49]. First of all, In_x_Al_1−x_As alloy band gap $E_{g}^{InAlAs}$ is controlled by the alloy composition *x* according to the quadratic approach [48]:

(1) $$E_{g}^{InAlAs}=x\cdot E_{g}^{InAs}+\left(1-x\right)\cdot E_{g}^{AlAs}-x\cdot \left(1-x\right)\cdot C_{InAlAs},$$

where $E_{g}^{InAs}$ and $E_{g}^{AlAs}$ are band gaps for InAs and AlAs, and *C*_InAlAs_ is the bowing parameter for the band gap of InAlAs.

Secondly, it is necessary to take into account the elastic deformations caused by the alloy composition deviation and their effect on the InAlAs band gap. According to the model-solid theory [49], the in-plane deformation for the elastically strained InAlAs layer matched with InP is

(2) $$\varepsilon_{xx}=\varepsilon_{yy}=\frac{a_{InP}-a_{InAlAs}}{a_{InAlAs}},$$

where $a_{InP}$ and $a_{InAlAs}$ are lattice parameters for InP and InAlAs, respectively.

The deformation along the growth direction is

(3) $$\varepsilon_{zz}=-2\varepsilon_{xx}\frac{C_{12}}{C_{11}},$$

where *C*_12_ and *C*_11_ are elastic constants for InAlAs.

The edge shift for the conduction and valence bands caused by hydrostatic deformation is

(4) $$H=\varepsilon_{xx}+\varepsilon_{yy}+\varepsilon_{zz}.$$

A change in the bandgap can be calculated by

(5) $$dE_{g}=H\cdot \left(a_{\Gamma }-a_{v}\right),$$

where *a*_r_ and *a*_v_ are deformation potentials for the electron and the hole, respectively. Material parameters for the InAlAs solid alloy were calculated according to the quadratic approach [48]. The values of the necessary parameters for InAs, InP and AlAs were taken from [48,50]. According to our calculations, the low-scale composition fluctuation magnitude is about 4%, whereas the average change in the composition for observed areas is about 0.7% for the structure grown at *T*_S_ = 535 °C. This value reduced to 0.3% at *T*_S_ = 505 °C (the PL band shift is about 6 meV).

Summarizing all of the above, we briefly list the main experimental results obtained:

1. The formation of the pits at the lattice-matched InAlAs/InP layer surface at *T*_A_ > 505 °C is observed.
2. Threading dislocations nucleated both in the InAlAs/InP heterointerface (for exact lattice-matched layers) and in the InAlAs bulk (for layers with the deviated alloy composition) are observed.
3. The dislocation outcrops are correlated with the edges of surface pits.
4. A ridge is formed along the perimeter of the pits.
5. The density of pits increases with the *T*_A_ and alloy composition deviation from the lattice-matched value.
6. The lateral sizes of the pits increase with the total InAlAs layer thickness in the case of the lattice-matched layer or the depth of dislocation nucleating in the InAlAs bulk in the case of the alloy deviation.
7. The depth of the pits increases with the total InAlAs layer thickness in the case of the lattice-matched layer or the depth of dislocation nucleating in the InAlAs bulk in the case of the alloy deviation and also with *T*_S_.
8. Clusters with deviated/shifted PL band peak energy are detected. The density and lateral sizes of the clusters are in good agreement with the corresponding parameters of the surface pits.
9. PL band peak deviation/shift corresponds to the local InAlAs alloy composition deviation with a magnitude of about 0.3–0.7%, increasing at/with *T*_S_.

## 4. Discussion

In the first part of this section, we discuss the reasons for and mechanisms of the formation of surface pits based on our experimental data. The second part of this section is devoted to the quantitative model that confirms our suggestion about the formation mechanisms for pits.

### 4.1. Surface Pit Formation Mechanisms

Our experimental data allow us to suggest that threading dislocations in the InAlAs layer are the main factor for the formation of surface pits. In addition to the direct dislocation–pit correlation demonstrated by the SEM data, this suggestion is confirmed by an increase in *N*_P_ observed depending on *T*_A_. It is known that the composition of an InAsP thin strained layer formed during InP substrate annealing depends on *T*_A_ [42]. An increase in the As fraction in this layer leads to an increase in lattice mismatching [48] and, consequently, the nucleation of dislocations with higher density [44,45,46,47]. Moreover, as shown by our data on the InAlAs layers with a slightly deviated composition, dislocations formed in the InAlAs bulk also have an effect on the surface relief. An increase in the lateral sizes of pits at an increase in the InAlAs layer thickness is in good agreement with this due to simple geometrical reasons for the branch divergence of threading dislocations.

The next step in elucidating the nature of surface pits is to discuss the mechanism of formation of pits in detail. We will focus on the fact of dislocation–cluster–pit correlation demonstrated by our experiments. Let us discuss the processes that may result in an alloy composition deviation during the InAlAs growth. Since the growth is performed in excess of As_4_, which is confirmed by the superstructure on the growth surface (2 × 4), the growth rate and composition of the InAlAs alloy are limited by group III materials, namely In and Al. A change in the correlation of the Al and In adatom incorporation rate has an effect on the alloy composition. The change in aluminum incorporation kinetics in the temperature range applied (up to 535 °C) can be neglected because the Al-As bond is much stronger than the In-As bond [14,51]. Therefore, only a change in the rate of indium incorporation can lead to the formation of the In-depleted clusters and pits on the surface. In the range of the temperatures considered, an intense In desorption from the growing InAlAs crystal surface is observed [52]. The main factor that has an effect on the In desorption rate is the growth temperature *T*_S_. As shown by our experimental data, a deviation in the cluster composition increases at *T*_S_, which confirms a desorption effect on the cluster composition. However, a non-local character of the temperature factor makes it insufficient for the description of pit formation. Another factor that may also have an effect on In adatoms incorporation kinetics is the strain. According to [53,54,55], in the case of III–V, the growth rate for adatom incorporation depends on crystal deformation. It is a well-known fact that the threading dislocation generates a local strain field [56]. Importantly, a sign of strain is opposite for different sides of the strain field in the vicinity of the dislocation. Thus, the dislocation strain field is a local factor that can help describe the formation of surface pits.

Let us discuss the factor of a dislocation strain field in detail. In the case of the tensile strain, the bonding energy of atoms decreases, whereas in the case of the compressive strain, it increases, on the contrary [55]. This means that depending on which side of the threading dislocation the In adatom is located, there is either a decrease or an increase in its binding energy. The tensile strain of the defect reduces the binding energy of the In adatom to the surface during the growth. This reduces the desorption barrier for In desorption and, consequently, reduces the rate of its incorporation, forming a pit/cluster. The compressive strain, on the contrary, increases the binding energy of the In adatom to the surface during growth. This effect forms a diffusion barrier for adatoms [54] and results in ridge formation. It should be noted that the surface diffusion of In adatoms averages the composition and growth rate of the ternary alloy. Therefore, to form a pit/cluster, it is necessary not only to reduce the desorption energy of indium adatoms in the cluster region but also to suppress the migration of indium adatoms from outside the pit. In our case, the presence of such a diffusion barrier reducing the migration of adatoms inside the pit is evidenced by the formation of a ridge along the perimeter of the pit. The ridge consists of many different surfaces with the direction strongly deviated from [001]. It is well known that the incorporation of adatoms proceeds more intensively on the surface with a developed relief, a high concentration of atomic steps and step breaks [57]. This not only suppresses the transfer of adatoms from a free surface to the pits but also stimulates ridge growth.

The increase in the depth of pits observed in our experiments at/with the total InAlAs layer thickness is in good agreement with the proposed model of pit formation due to the direct relationship between pit development and total growth time. In order to confirm the model, we suggest a quantitative description of pit formation.

### 4.2. Modeling of Pit Formation

In order to verify the proposed mechanism of pit/cluster formation and to estimate the magnitude of the barrier reduction for In desorption at the defect, the experimental dependence of the pit depth (*H*) and the In fraction change (Δ*x*) on *T*_S_ was approximated by expressions in the framework of the following model. Since the growth is performed in As_4_ excess conditions, the growth rate for In_x_Al_1−x_As (*V*_InAlAs_) is ruled by the total growth rate of binary compounds as follows:

(6) $$V_{InAlAs}=V_{InAs}+V_{AlAs}$$

where *V*_InAs_ and *V*_AlAs_ are the growth rates of InAs and AlAs, respectively. According to (6), the alloy composition can be calculated as

(7) $$x=\frac{V_{InAs}}{V_{InAs}+V_{AlAs}}$$

Then, the alloy composition difference between the clusters and free surface is

(8) $$\Delta x=x^{cluster}-x=\frac{V_{InAs}^{cluster}}{V_{InAs}^{cluster}+V_{AlAs}^{cluster}}-\frac{V_{InAs}}{V_{InAs}+V_{AlAs}}$$

where *x*^cluster^ is the alloy composition in the cluster observed, and $V_{InAs}^{cluster}$ and $V_{AlAs}^{cluster}$ are binary growth rates in the cluster.

As suggested above, there is no migration of indium adatoms between the cluster and the free surface of the film due to ridge formation. This allows us to calculate the pit depth as

(9) $$H=W_{InAlAs}^{cluster}-W_{InAlAs}$$

where $W_{InAlAs}^{cluster}$ and $W_{InAlAs}$ are total layer thicknesses for the cluster and the free surface, respectively.

Layer thickness depends on the growth rate, interplanar spacing for (001) planes *d*_001_ for the InAlAs alloy and total growth time *t*_g_:

(10) $$\begin{array}{c} W_{InAlAs}^{cluster}=V_{InAlAs}^{cluster}\cdot d_{001}\cdot t_{g}\\ W_{InAlAs}=V_{InAlAs}\cdot d_{001}\cdot t_{g} \end{array}$$

Thus, the pit depth can be expressed as

(11) $$H=(V_{InAs}^{cluster}+V_{AlAs}^{cluster}-V_{InAs}-V_{AlAs})\cdot d_{001}\cdot t_{g}$$

According to our model, the temperature dependence of the growth rate of binary compounds outside/inside the cluster can be represented as the following expressions, taking into account only the adsorption/desorption processes of group III elements:

(12) $$\begin{array}{l} V_{InAs}=F_{In}\cdot (K_{In}-K_{d\_In}(T));\;\\ V_{InAs}^{cluster}=F_{In}\cdot (K_{In}-K_{d\_In}^{cluster}(T));\\ V_{AlAs}=F_{Al}\cdot (K_{Al}-K_{d\_Al}(T));\\ V_{AlAs}^{cluster}=F_{Al}\cdot (K_{Al}-K_{d\_Al}^{cluster}(T)); \end{array}$$

where *K_In_* and *K_Al_*—adsorption coefficients of In and Al equal to 1 under As excess conditions; *F_i_*—the normalized measured flux of incident In (~0.33 ML/s) or Al (~0.3 ML/s) cations; *K_d_In_*/$K_{d\_In}^{cluster}$ and *K_d_Al_*/$K_{d\_Al}^{cluster}$—In and Al desorption coefficients in/out cluster, respectively. As we discussed earlier, Al desorption is very weak at the temperatures used, which allows us to neglect the difference between *K_d_Al_* and $K_{d\_Al}^{cluster}$. Therefore, pit depth can be presented as

(13) $$H=(V_{InAs}^{cluster}-V_{InAs})\cdot d_{001}\cdot t_{g}$$

The cation desorption constant was described by the Arrhenius equation as follows:

(14) $$K_{d\_In/Al}=K_{0}\cdot \exp\left(-\frac{E_{A\_In/Al}}{kT}\right)$$

where *K*_0_ is the pre-exponential factor equal to $\frac{kT}{2\pi \hbar }$, which is close to 10^13^ s^−1^ at *T* = 505 °C and caused by atom thermal oscillation [58], and *E_A_In/Al_* is the energy barrier of the In/Al desorption reaction.

**Figure 6:**
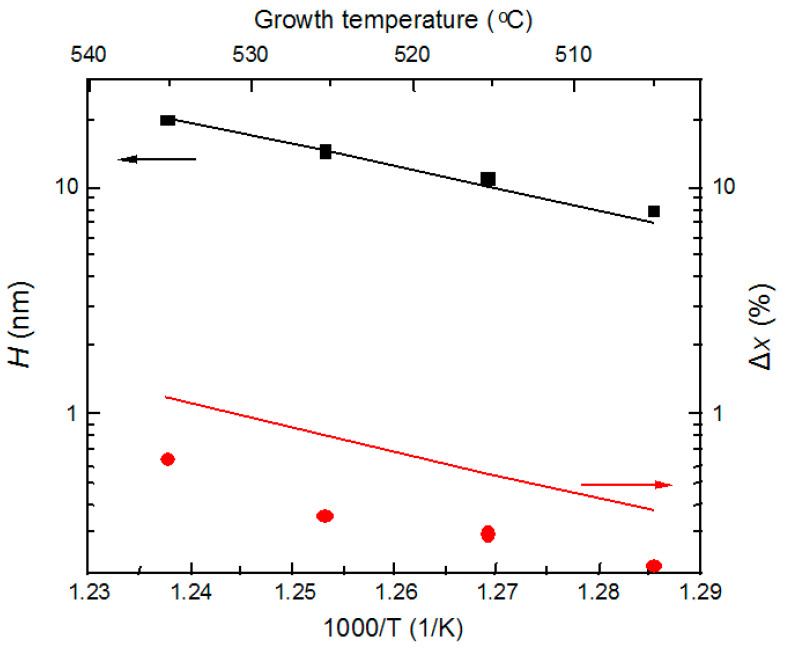
Experimental and calculated temperature dependencies of the pit depth (black square) and In depletion (red circle) for lattice-matched 1 μm thick InAlAs layers.

In order to link our model to the experimental data of *H*(*T*_S_) and Δ*x*(*T*_S_), we performed the variation of *E_A_In/Al_* for clusters and free surfaces using the least square deviation method. As one can see from Figure 6, the proposed model of pit/cluster formation qualitatively describes the experimental results. Some differences in the calculated and experimental dependencies for the cluster composition can be explained by the fact that the micro-FL experiment gives lower composition values due to the constant error caused by the relatively large size of the laser spot (>1 μm) compared to the cluster size. The decrease in the desorption energy of indium on the defect we found was of the order of 27–33 meV, with the desorption energy of In and Al equal to 2.0 and 2.5 eV, respectively. The In desorption energy obtained is close to the literature data of 1.9–2.2 eV [55,59,60]. There is little information on the reduction of desorption barriers for In on lattice defects in InAlAs/InP in the literature. Nevertheless, the desorption barrier reduction on mismatch dislocations in InAsP is known to be in the order of 20–40 meV [61], which almost coincides with the values obtained.

## 5. Conclusions

The pit formation on the surface of the lattice-matched InAlAs layer grown on the InP (001) substrate has been detected. The investigation of the surface morphology allows us to clarify the dependencies of the pit parameters (*N*_P_, *L*, *H*) upon the growth parameters (*T*_A_, *T*_S_, *D*). It has been determined that the formation of pits occurs only at *T*_A_ > 505 °C. The depth of pits *H* is controlled by *T*_S_ and *D*, whereas the lateral sizes of pits *L* are controlled by *D*. The analysis of cross-section images shows that the surface pits are correlated with the threading dislocation, nucleated both in a thin InAsP layer on the InAlAs/InP interface and in the bulk of the InAlAs layer. In addition, the formation of the surface clusters with a deviated alloy composition has been detected. Cluster lateral sizes and density values are close to the pit parameters, which allows us to suggest a correlation between these objects.

Based on the experimental data, we propose a description model for the formation of pits. In the framework of this model, a dislocation-assisted strain field leads to an increase in In desorption, which results in the formation of clusters and surface pits. The quantitative analysis that takes into account the temperature dependencies of the desorption coefficient for In has confirmed this due to the comparison of the calculated and experimental dependencies of *H* and the alloy composition deviation values. It has been found that a decrease in the desorption barrier value of In adatoms in the vicinity of dislocation outcrops is about 27–33 meV.

## Data Availability

Data are available from the authors on request.
